# Antineutrophilic Cytoplasmic Antibody Positive Vasculitis Associated with Methimazole Use

**DOI:** 10.1155/2015/530319

**Published:** 2015-04-28

**Authors:** Deep Shikha, Jonathan Harris, Christine Resta, Patricia Park

**Affiliations:** ^1^Department of Endocrinology, SUNY Downstate Medical Center, Brooklyn, NY 11203, USA; ^2^Department of Pathology, Maimonides Medical Center, Brooklyn, NY, USA; ^3^Department of Endocrinology, Maimonides Medical Center, Brooklyn, NY, USA

## Abstract

ANCA-associated vasculitis (AAV) is a rare and potentially life threatening complication associated with antithyroid drug use. It is more commonly reported with propylthiouracil, with fewer cases reported with methimazole use. We present the case of a 55-year-old man with toxic multinodular goiter which was treated with methimazole for 6 months. He developed ANCA positive leukocytoclastic vasculitis with hemorrhagic and necrotic bullous lesions of lower extremities. The vasculitis was initially thought to be secondary to recent cephalosporin use; however, the skin lesions progressed despite stopping the cephalosporin and treatment with steroids, and he developed osteomyelitis. His vasculitis resolved after cessation of methimazole use. This case highlights the importance of careful monitoring for variable manifestations of AAV in patients treated with methimazole.

## 1. Introduction

Methimazole (MMI) and propylthiouracil (PTU) are thionamide drugs, which are commonly used as first-line therapy in the treatment of hyperthyroidism due to Grave's disease and toxic nodular goiter in the United States. These medications are not without risk, however, and are associated with potential adverse reactions such as fever, rash, agranulocytosis, and hepatitis. These reactions usually occur within the first few months of initiating treatment [1], although agranulocytosis can occur idiosyncratically at any time during treatment. ANCA positive vasculitis is a serious but lesser known complication of thionamides. Despite being previously described in the literature, there is a lower incidence of reported ANCA positive vasculitis with MMI use as compared to PTU [2, 3]. We report a patient who developed ANCA positive leukocytoclastic vasculitis after six months of MMI treatment.

## 2. Case

A 55-year-old male was diagnosed with hyperthyroidism by his primary care physician. Thyroid sonogram showed a multinodular goiter. FNA biopsies of the dominant nodules were benign, and he was started on methimazole 20 mg twice a day for toxic nodular goiter. Six months later, he presented to the emergency department with bilateral lower extremity pain, redness, and swelling. He was diagnosed with cellulitis and discharged home on oral cephalexin; however, his lower extremity lesions progressed over the next month, and he was admitted to the hospital for further management.

**Figure 1:**
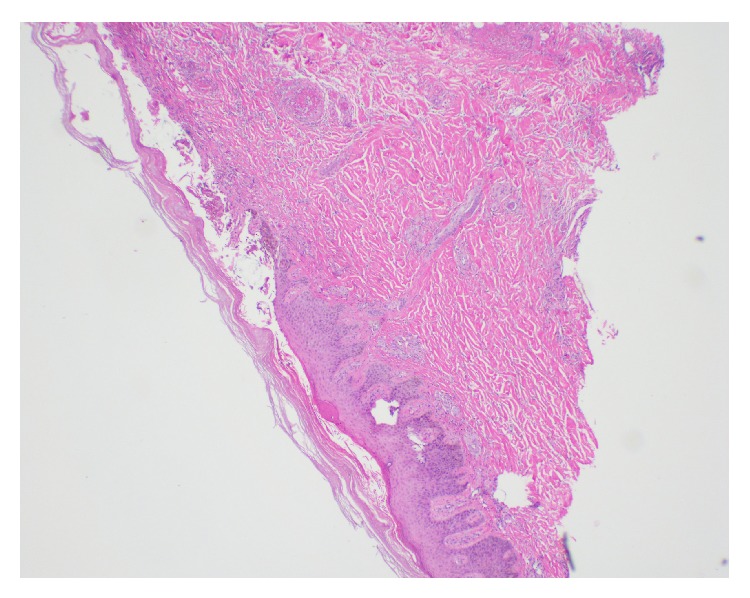
Skin biopsy in low power field showing leukocytoclastic vasculitis.

**Figure 2:**
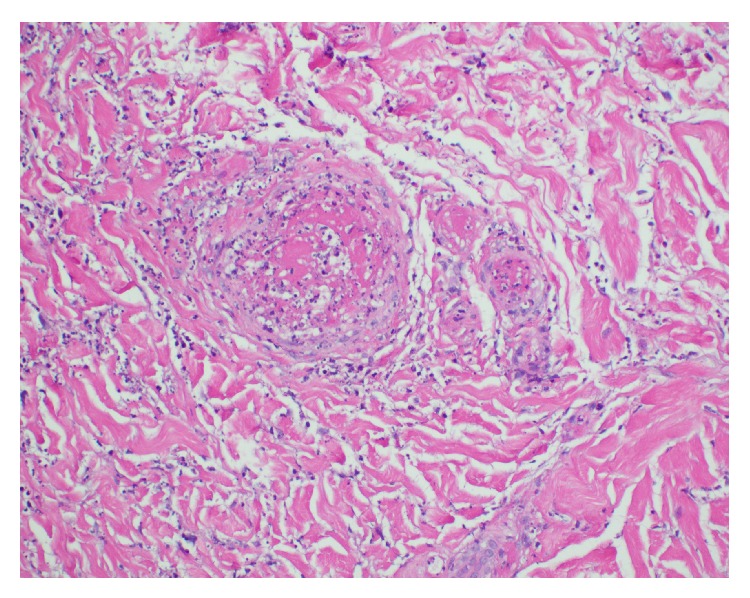
Skin biopsy in high power field showing leukocytoclastic vasculitis.

During that admission, the patient was noted to have hemorrhagic and necrotic bullous lesions on the anterior aspect of the bilateral lower legs and dorsal aspect of the feet. Laboratory data showed elevated C-reactive protein suggestive of an inflammatory reaction, but without leukocytosis or eosinophilia. He had normal levels of rheumatoid factor, ribonucleoprotein antibody, and Sjogren SSA and SSB antibodies. Serum complement C3 and C4 levels were high; C3 was 180 mg/dL (<90 mg/dL) and C4 was 50 mg/dL (6–47 mg/dL). Antinuclear antibody (ANA) was positive in titres of 1 : 80 with a speckled pattern. ANCA screen as measured with indirect immunofluorescence was positive for p-ANCA and detected high MPO antibodies at 5.6 AI (normal < 1 AI). Work-up for HIV, hepatitis B, and hepatitis C was negative. Urinalysis was unremarkable. Skin biopsy of the lesions revealed leukocytoclastic vasculitis with fibrin thrombi. No immune deposits were detected (Figures 1 and 2).

Based on this work-up, the vasculitis was attributed to cephalexin. The patient was treated with high dose prednisone for 2 weeks in the hospital and discharged home with an additional 2 weeks of tapering glucocorticoids. He presented again 2 months later with persistent bilateral lower extremity skin lesions and suppurative discharge from the left foot. MRI and bone biopsy were consistent with acute osteomyelitis.

The endocrinology team was consulted during this readmission because of high TSH while being on methimazole. On examination, he had no lid lag or exophthalmos. Thyroid was nodular and enlarged about three times the normal size, with left lobe bigger than right. CXR showed an enlarged left thyroid lobe deviating the upper trachea to the right side. Thyroid antibodies were not elevated: thyroid peroxidase antibody was 14 IU/mL (<35 IU/mL), thyroglobulin antibody was <20 IU/mL (<20 IU/mL), and thyroid stimulating immunoglobulin was 125% (<140%).

The lower extremity lesions did not resolve despite stopping cephalexin and completing month-long course of steroids; therefore, we considered the possibility of methimazole-induced leukocytoclastic vasculitis. Methimazole was discontinued. We then recommended total thyroidectomy for definitive management of a toxic multinodular goiter that was also causing tracheal deviation. Surgical pathology showed nodular hyperplasia with focal Hurthle cell features and calcifications with ossification. He was started on levothyroxine replacement therapy and antibiotics for osteomyelitis and discharged home. On 1-month follow-up in clinic, the patient's skin lesions were largely resolved and he was clinically well.

## 3. Discussion

ANCA-associated vasculitis (AAV) is a group of small vessel vasculitides that consist of autoantibodies directed against the lysosomal enzymes of neutrophils. These autoantibodies are divided into two main groups: cytoplasmic (c-ANCA) which confers antigen specificity for proteinase 3 and is associated with Wegener's granulomatosis and perinuclear (p-ANCA) which reacts against myeloperoxidase (MPO) and is mainly associated with microscopic polyangiitis (MPA) and Churg-Strauss syndrome. AAV may cause a variety of constitutional symptoms including fever, myalgia, arthralgia, and flu like syndrome. Multisystem involvement can be seen, with the kidneys most commonly affected followed by skin and respiratory tract [3]. Vessels in the joints, eyes, skeletal muscle, gastrointestinal tract, and peripheral nerves may also be involved. The most common cutaneous lesion is leukocytoclastic vasculitis, which preferentially affects the lower extremities [4].

AAV in association with antithyroid drugs is a relatively uncommon entity, and the pathogenesis of vasculitis associated with ATDs is not well understood. There have been reports of cutaneous and systemic AAV, with most of the literature describing p-ANCA vasculitis in the setting of PTU use, particularly in Asian individuals [2–10]. It has been postulated that PTU binds to myeloperoxidase and alters its structure, leading to formation of autoantibodies in susceptible individuals [11]. There are no published data that suggest that methimazole can also alter the configuration of myeloperoxidase. Data from Gao et al. [12] indicate that activated neutrophils produce increased amounts of myeloperoxidase which oxidize the thionamides into reactive intermediates. These intermediates then activate immunocompetent cells such as lymphocytes via covalently binding to self-proteins, leading to production of MPO-ANCA and hence causing vascular injury.

AAV is a rare complication of MMI use, with few cases described in the literature. The first case of MMI-induced AAV was described by Kawachi et al. in 1995 [6]. AAV is also less frequently described in toxic MNG compared to Grave's disease. In 1996, Gunton et al. assembled 27 cases of ATD-induced ANCA positive vasculitis in which only one case was related to MMI and only 1 case had underlying toxic MNG as the etiology of hyperthyroidism [2].

Noh et al. have reported that the incidence for PTU-related AAV is about 39 times that for MMI [3]. Despite being associated with long term antithyroid drug treatment, with median onset time 42 months, it can also occur within a few months of starting the treatment. MPO-AAV can occur even at low doses for both MMI and PTU. It is reported to occur more frequently in women, although this may just reflect the female preponderance of thyroid disease. Interestingly, the appearance of p-ANCA antibodies does not always predict the development of clinical vasculitis [13], and there is also no correlation between the MPO-ANCA titer and the severity of vasculitis [3].

Importantly, p-ANCA-associated vasculitis improves and has a good prognosis if antithyroid drugs are discontinued. Some patients may require steroids and/or immunosuppressive drugs depending upon the severity of the disease. Serious complications (pulmonary hemorrhage, acute kidney failure, and nonhealing ulcers) have been reported when ATDs have been continued due to unawareness of this uncommon adverse reaction.

Our patient had leukocytoclastic vasculitis with cutaneous lesions and no systemic manifestations. His vasculitis was initially thought to be secondary to cephalosporin use, but the skin lesions did not heal despite stopping cephalosporin and completing a course of prednisone. The patient in fact had progression of the lower extremity ulcers and developed osteomyelitis. After MMI was discontinued, the skin lesions resolved. This clinical course along with the presence of high p-ANCA titres suggested that the biopsy-proven leukocytoclastic vasculitis was likely an AAV due to MMI use. We did not recommend PTU therapy after stopping MMI due to the higher reported incidence of vasculitis with PTU use and reports of cross reactivity between PTU and MMI [14] in AAV.

In conclusion, we suggest that clinicians be aware of this uncommon adverse reaction of MMI treatment. Patients treated with MMI should be carefully monitored for manifestations of AAV such as fever, skin involvement, myalgias, arthralgias, glomerulonephritis, and pulmonary hemorrhage, regardless of the period of administration of MMI. Early recognition of this serious adverse effect is crucial as immediate cessation of MMI is needed, along with possible administration of corticosteroids, in order to prevent progression of the disease and its serious sequelae.
